# Prevalence and coexistence of type 2 inflammatory diseases

**DOI:** 10.1002/clt2.12376

**Published:** 2024-06-19

**Authors:** Toni Mora, Irene Sánchez‐Collado, Rosa Muñoz‐Cano, Paula Ribó, Paloma I. Palomo‐Jiménez, Joaquim Mullol, Antonio Valero

**Affiliations:** ^1^ Research Institute for Evaluation and Public Policies Universitat Internacional de Catalunya (UIC) Barcelona Catalonia Spain; ^2^ Allergy Department Universitat de Barcelona Hospital Clinic Barcelona Catalonia Spain; ^3^ Institut d’Investigacions Biomèdiques August Pi i Sunyer Barcelona Catalonia Spain; ^4^ RICORS Instituto de Salud Carlos III Madrid Spain; ^5^ CIBER of Respiratory Diseases (CIBERES) Instituto de Salud Carlos III Madrid Spain; ^6^ Sanofi Madrid Spain; ^7^ Rhinology Unit and Smell Clinic ENT Department Hospital Clinic Universitat de Barcelona Barcelona Catalonia Spain

**Keywords:** asthma, atopic dermatitis, epidemiology, Nasal polyps, population‐based, prevalence Catalonia, type 2 inflammatory diseases

## Abstract

**Background:**

Type 2 inflammation has been described as a pathophysiological basis common to some diseases, such as atopic dermatitis (AD), chronic rhinosinusitis with nasal polyps, and asthma (CRSwNP).

**Objective:**

The present study used population‐based prevalence in Catalonia to analyse the coexistence of type 2 inflammatory diseases in patients primarily diagnosed with the above mentioned conditions.

**Results:**

We found a high degree of coexistence of type 2 inflammatory diseases among these patients, with the prevalence being higher in the severe forms, except for AD. For the severe forms of primary diseases, the proportion of patients with coexisting type 2 inflammatory diseases (severe or non‐severe) was 16.2% for AD, 19.8% for asthma, and a striking 62.4% for CRSwNP. This patient population has the highest proportion of coexisting type 2 inflammatory diseases, both severe (48.9%) and non‐severe (13.5%).

**Conclusion:**

Our findings have significant implications for the management of patients with AD, asthma, and CRSwNP.

## 1 INTRODUCTION AND METHODS

Type 2 (T2) inflammation has been described as a pathophysiological basis common to some diseases such as atopic dermatitis (AD), chronic rhinosinusitis with nasal polyps (CRSwNP), and asthma.^1^ Different studies have shown that T2 inflammatory (T2i) diseases frequently coexist in the same patient,^2^, ^3^, ^4^ directly impacting disease severity, quality of life and costs while making it more difficult to control the disease.^5^

The present study has focused on analysing the coexistence of T2 inflammatory diseases in patients primarily diagnosed with AD, asthma or CRSwNP using population‐based prevalence in Catalonia. Methods and datasets were fully explained in refs. [6, 7, 8]. Our epidemiological study used a retrospective large‐scale population‐based database covering 2013–2017. We considered all residents in Catalonia, including those covered by the statutory National Health Service (NHS) and included in the Agency for Health Quality and Assessment of Catalonia database. The inclusion criteria used are based on the diagnosis by medical records (ICD‐9‐CM codes) at any care level covered by the NHS (primary, hospital, and emergency care) at any time point from January 2013 until December 2017 (different follow‐up periods for patients in the dataset). Disease severity was defined using the following observations, as in refs. [6, 7, 8]: For AD: (1) prescription of immunosuppressant agents (cyclosporin, azathioprine, cyclophosphamide, methotrexate, alitretinoin, mycophenolic acid, interferon alpha‐2a, interferon alpha‐2b) at least once during the last 2 years; or (2) one or more hospitalisations/emergencies over the last 2 years with AD as a first diagnosis. For asthma: (1) high dose intake of inhaled corticosteroids for at least 6 months over the last year or at least 12 months over the last 2 years (consecutive or not); or (2) treatment with biologics over the last 2 years; or (3) intake of systemic corticosteroids (SCS) for more than 6 months over the last year or more than 12 months over the last 2 years. Finally, for CRSwNP: (1) SCS intake over the last 2 years above the annual minimum dose calculated threshold for each SCS in any of the 2 years; or (2) endoscopic sinus surgery over the period for which we have information (2010–2017).

Our main objectives were to assess: (1) the prevalence of comorbid T2 inflammatory diseases for each primary disease (AD, asthma, or CRSwNP) and their degrees of severity (severe and non‐severe); and (2) the prevalence of comorbid T2 inflammatory diseases (severe and non‐severe) for each primary disease (AD, asthma, or CRSwNP) in each degree of severity (severe and non‐severe).

## 2 RESULTS

The Ethical Review Board in Hospital Clínic (Spain) approved the study. Out of the total Catalan population in 2017 (7,555,830 inhabitants), 871,309 patients received a primary diagnosis of AD, 473,737 of asthma and 30,819 of CRSwNP from 2013 to 2017. Among T2 inflammatory diseases, AD had the highest prevalence in the general population (11.53%), followed by asthma (6.3%) and CRSwNP (0.41%) (Table 1). All analysed T2 inflammatory diseases showed statistically significant lower prevalence in their severe form relative to non‐severe: AD (0.22%), asthma (0.39%), and CRSwNP (0.10%), with asthma as the T2 inflammatory disease with the highest prevalence in its severe form. CRSwNP was the disease with the highest proportion of patients in its severe form (24%) (Table 1). We observed high comorbidity of severe and non‐severe forms for each primary disease in the prevalence analysis of other comorbid T2 inflammatory diseases. Asthma was the most common (40,06%) in patients with primary CRSwNP. Except for AD, the prevalence of comorbid T2 inflammatory disease was more frequent in severe forms, with asthma also the most prevalent disease in patients with severe CRSwNP (52.85%) (Table 1). When comorbid T2 inflammatory diseases, either non‐severe or severe, were analysed for each primary severe diagnosis (AD, asthma and CRSwNP) (Table 2), it was observed that (i) in severe AD, the most common comorbid disease was asthma; both non‐severe (12.95%) and severe (2.18%); (ii) in severe asthma, the most common comorbid diseases were non‐severe AD (13.62%) and severe CRSwNP (2.66%); and (iii) in severe CRSwNP, the most common comorbid disease was also non‐severe (34.42%) and severe (10.58%) asthma.

**TABLE 1:** Population prevalence of T2 inflammatory diseases based on severity and coexistence of other T2 inflammatory pathologies.

	Prevalence based on diagnosis (%)	AD coexistence (%)	Asthma coexistence (%)	CRSwNP coexistence (%)
AD; *N* = 871,309	11.53	‐	9.60	0.62
Non‐severe *N* = 854,711 (98.1%)	11.31^a^		9.49^a^	0.61
Severe *N* = 16,598 (1.9%)	0.22		15.44	1.07
Asthma; *N* = 473,737	6.30	17.66	‐	2.61
Non‐severe *N* = 444,306 (93.8%)	5.90^a^	17.79^a^		2.45^a^
Severe *N* = 29,431 (6.2%)	0.39	15.65		4.96
CRSwNP; *N* = 30,819	0.41	17.48	40.06	‐
Non‐severe *N* = 23,427 (76%)	0.31^a^	17.50	36.03^a^	
Severe *N* = 7392 (24%)	0.10	17.41	52.85	

Abbreviations: AD, atopic dermatitis; CRSwNP, chronic rhinosinusitis with nasal polyps.

^a^ Statistical significance at 1% between non‐severe and severe within each disease.

**TABLE 2:** Coexistence of severe and non‐severe T2 inflammatory diseases in patients with severe forms of the main diagnosis (AD, asthma and severe CRSwNP).

		Coexistence severe T2					Coexistence non‐severe T2			
	NO T2	Only AD	Only asthma	Only CRSwNP	1 severe + 1 non severe	Both severe	Only AD	Only asthma	Only CRSwNP	Both non‐severe
	*N* (%)	*N* (%)	*N* (%)	*N* (%)	*N* (%)	*N* (%)	*N* (%)	*N* (%)	*N* (%)	*N* (%)
Severe AD (*N* = 16,598)	13,909 (83.80)	‐	362 (2.18)^a^	58 (0.35)^a^	16 (0.10)^a^	12 (0.08)^a^	‐	2149 (12.95)^a^	68 (0.41)^a^	24 (0.14)^a^
Severe asthma (*N* = 29,431)	23,601 (80.20)	362 (1.2)^a^	‐	782 (2.66)^a^	140 (0.48)^a^	12 (0.04)^a^	4008 (13.62)^a^	‐	441 (1.50)^a^	85 (0.29)^a^
Severe CRSwNP (*N* = 7392)	2779 (37.60)	58 (0.78)^a^	782 (10.58)^a^	‐	144 (1.95)^a^	12 (0.16)^a^	648 (8.77)^a^	2544 (34.42)^a^	‐	425 (5.75)^a^

*Note*: Overall analysed population, *N* = 7,555,830.

Abbreviations: AD, atopic dermatitis; CRSwNP, chronic rhinosinusitis with nasal polyps.

^a^ Statistical significance at 1% for each prevalence compared to the NO T2 category.

**FIGURE 1:**
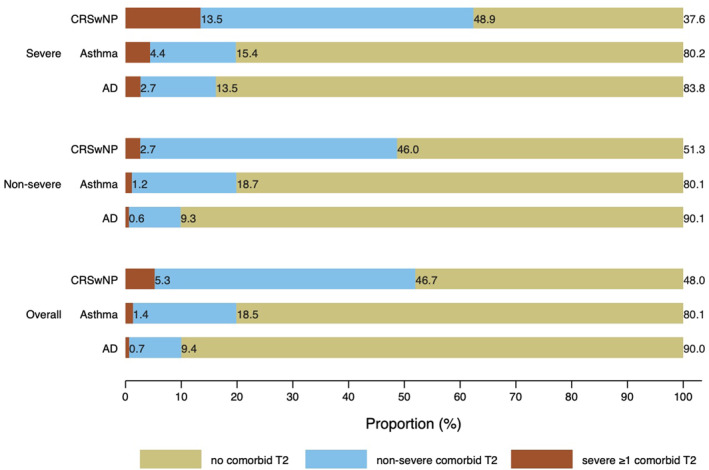
The proportion of patients with comorbid T2 inflammatory diseases according to severity and associated comorbidities. AD, atopic dermatitis; CRSwNP, chronic rhinosinusitis with nasal polyps.

Finally, we also analysed the coexistence of T2 inflammatory disease, considering the association of severity degrees of the primary diagnosis with the comorbid T2 inflammatory diseases. For the severe forms of primary diseases, the proportion of patients with comorbid diseases (severe or non‐severe) was 16.2% for AD, 19.8% for asthma, and 62.4% for CRSwNP, the latter being the patient's population with the highest proportion of coexisting T2 inflammatory diseases, both severe (48.9%) and non‐severe (13.5%) (Figure 1). For the non‐severe forms of primary diseases, the proportion of patients with comorbid diseases (severe or non‐severe) was 9.9% for AD, 19.9% for asthma and 48.7% for CRSwNP.

## 3 CONCLUSION

The results of this population‐based study evidence a high degree of coexistence of T2 inflammatory diseases among patients with AD, asthma, and CRSwNP, with coexistence being higher in the severe forms, except for those in AD. In fact, for the severe forms of primary diseases, the proportion of patients with coexisting T2 inflammatory diseases (severe or non‐severe) was 16.2% for AD, 19.8% for asthma, and 62.4% for CRSwNP, the latter being the patient's population with the highest proportion of coexisting T2 inflammatory diseases, both severe (48.9%) and non‐severe (13.5%). A greater comprehension of the underlying T2 inflammation, common to several comorbid T2 inflammatory diseases, may help health professionals optimise and simplify treatments to achieve their control and severity while improving patients' quality of life.

## AUTHOR CONTRIBUTIONS

**Toni Mora**: Conceptualization; investigation; funding acquisition; writing—original draft; methodology; validation; visualization; writing—review and editing; software; formal analysis; project administration; data curation; supervision. **Irene Sánchez‐Collado**: Conceptualization; data curation; methodology. **Rosa Muñoz‐Cano**: Conceptualization; investigation. **Paula Ribó**: Conceptualization; investigation. **Paloma I. Palomo‐Jiménez**: Conceptualization; investigation; methodology; writing—review and editing; validation. **Joaquim Mullol**: Conceptualization; investigation; supervision; validation. **Antonio Valero**: Supervision; validation; methodology; conceptualization; investigation; project administration.

## CONFLICT OF INTEREST STATEMENT

PPJ is a Sanofi employee. The rest of the authors received specific funding for developing this work from the International University of Catalonia (UIC) Real‐World Evidence Chair. There are no patents, products in development or marketed products to declare. The authors of this manuscript have no relevant financial or other relationships to disclose. JM is or has been a member of national and international scientific advisory boards, received fees for lectures and consulting, and grants for research projects or clinical trials from AstraZeneca, Genentech‐Roche, GSK, LETI, Lilly, Menarini, MSD, Mitsubishi‐Tanabe, NOUCOR/Uriach, Novartis, OPTINOSE, Procter and Gamble, Regeneron Pharmaceuticals Inc., Sanofi, UCB Pharma, and Viatris/MEDA Pharma.

4

## Data Availability

The datasets analysed during the current study are not publicly available because they correspond to administrative registers under an agreement which does not allow researchers to share the database with any other third party.
